# Editorial Notes, News and Miscellany

**Published:** 1914-01

**Authors:** 


### Editorial Notes, News and Miscellany.

The Great Neurologist and Author is dead: Dr. S. Weir Mitchell, aged 85.

---

For $1.60 (the price of the book and postage), I will send a copy of Dr. Bruno, and the "Red Back" one year.

---

Dr. Jacoby testifies (*N. Y. Med. Journal*) that he has been cured of epithelioma of the face, by three applications of radium.

---

E. H. Carey, Jr. (son of Dr. and Mrs. E. H. Carey, of Dallas, born Dec. 10th) sends Christmas Greetings to the "Red Back." Same to you, my son. Bully boy!

---

Of Age and Can Vote Now.—Whoop-la! Abbott's *American Journal of Clinical Medicine* will celebrate its 21st anniversary with a special number of great excellence for January; and has prepared a program that, metaphorically, makes one's mouth water, in anticipation. I wish I had room to produce it but I haven't. Send for a specimen copy and subscribe.

---

Things Happen so often and so fast that it is hard to keep up with them. I think, one day "I'll write an editorial on this," but something happens and knocks it out. It is sorterlike Opie Reed said of the autos when they first arrived, "Before I could say 'here she comes,' I have to say, 'there she goes.'" But one thing: I'm tired of eugenics and heredity, likewise 'alcohol.' One fellow got mad and quit because in the last issue I had an editorial in which I begged to differ with Thomas Jefferson about prohibition.

---

A Successful Medical Practitioner of many years standing makes the following statement:

"There are a large majority of combinations which extemporaneous pharmacy cannot prepare properly; and I know that through the dishonesty, ignorance, or indifference of many retail druggists we are not able to get on prescriptions the very best drugs; hence it is to the manufacturing pharmacist, whose best interest lies in the purity and uniformity of this product, that we look for our most reliable remedies.

"I endorse worthy proprietaries, but I most heartily condemn the great tendency of manufacturing pharmacists to foist upon the profession and public cheap imitations of standard preparations."

---

Mrs. Daniel's Work Highly Commended.—Prof. Ivy, of Sherman, writes: "I think your editorial in the *White Ribbon* on 'A Single Moral Standard,' was superb."

Mrs. J. H. Potter, of El Paso, says: "I desire to express my approbation of your article: 'Shall We Have One Standard of Morals; What Shall the New Standard Be?' I wish that that article might be copied in every paper in the land. Good men already know and acknowledge its truth, and the balance must be educated."

